# Engineering a wild-type diploid *Saccharomyces cerevisiae* strain for second-generation bioethanol production

**DOI:** 10.1186/s40643-016-0126-4

**Published:** 2016-11-24

**Authors:** Hongxing Li, Yu Shen, Meiling Wu, Jin Hou, Chunlei Jiao, Zailu Li, Xinli Liu, Xiaoming Bao

**Affiliations:** 1 State Key Laboratory of Microbial Technology, Shandong University, Shan Da Nan Road 27, Jinan, 250100 China; 2 Shandong Provincial Key Laboratory of Microbial Engineering, Qi Lu University of Technology, Jinan, 250353 China

**Keywords:** Xylose-specific transporter, Xylose isomerase, Synchronous utilization of xylose and glucose, Lignocellulosic ethanol, Budding yeast

## Abstract

**Background:**

The cost-effective production of second-generation bioethanol, which is made from lignocellulosic materials, has to face the following two problems: co-fermenting xylose with glucose and enhancing the strain’s tolerance to lignocellulosic inhibitors. Based on our previous study, the wild-type diploid *Saccharomyces cerevisiae* strain BSIF with robustness and good xylose metabolism genetic background was used as a chassis for constructing efficient xylose-fermenting industrial strains. The performance of the resulting strains in the fermentation of media with sugars and hydrolysates was investigated.

**Results:**

The following two novel heterologous genes were integrated into the genome of the chassis cell: the mutant *MGT05196* ^N360F^, which encodes a xylose-specific, glucose-insensitive transporter and is derived from the *Meyerozyma guilliermondii* transporter gene *MGT05196*, and Ru-*xylA* (where Ru represents the rumen), which encodes a xylose isomerase (XI) with higher activity in *S. cerevisiae*. Additionally, endogenous modifications were also performed, including the overproduction of the xylulokinase Xks1p and the non-oxidative PPP (pentose phosphate pathway), and the inactivation of the aldose reductase Gre3p and the alkaline phosphatase Pho13p. These rationally designed genetic modifications, combined with alternating adaptive evolutions in xylose and SECS liquor (the leach liquor of steam-exploding corn stover), resulted in a final strain, LF1, with excellent xylose fermentation and enhanced inhibitor resistance. The specific xylose consumption rate of LF1 reached as high as 1.089 g g^−1^ h^−1^ with xylose as the sole carbon source. Moreover, its highly synchronized utilization of xylose and glucose was particularly significant; 77.6% of xylose was consumed along with glucose within 12 h, and the ethanol yield was 0.475 g g^−1^, which is more than 93% of the theoretical yield. Additionally, LF1 performed well in fermentations with two different lignocellulosic hydrolysates.

**Conclusion:**

The strain LF1 co-ferments glucose and xylose efficiently and synchronously. This result highlights the great potential of LF1 for the practical production of second-generation bioethanol.

**Electronic supplementary material:**

The online version of this article (doi:10.1186/s40643-016-0126-4) contains supplementary material, which is available to authorized users.

## Background

Bioethanol produced from lignocellulose, i.e., second-generation bioethanol, is benefit for a sustainable energy supply. Its cost-effective production process depends on the complete and rapid utilization of all the sugars in the hydrolysates derived from lignocellulosic raw materials (Ko et al. 2016; Moysés et al. 2016; Peng et al. 2012; Zhou et al. 2012). The budding yeast *Saccharomyces cerevisiae* is a prominent microorganism that has traditionally been used in industrial bioethanol production because of its numerous inherent advantages (Demeke et al. 2013). However, this natural ethanol producer must overcome at least two new challenges when the fermentation substrate is lignocellulosic hydrolysates, rather than starch-based materials. First, natural *S. cerevisiae* cannot effectively metabolize pentose sugars, particularly d-xylose, the second most abundant sugar in lignocellulosic materials because it lacks an effective initial metabolic pathway (Hahn-Hägerdal et al. 2007; van Maris et al. 2006). Second, the individual and synergistic negative interactions derived from the numerous inhibitory compounds that are formed during the pretreatment process and the hydrolytic release of sugars exert serious negative effects on the fermentation performance of *S. cerevisiae* (Ko et al. 2016; Palmqvist and Hahn-Hägerdal 2000). Therefore, for the economically viable and sustainable production of lignocellulosic bioethanol, it is necessary to confer the capacity to co-ferment glucose and xylose on an *S. cerevisiae* strain and to enhance its resistance to harsh production environments (Demeke et al. 2013; Li et al. 2015; Sharma et al. 2016).

The genetic background of the host strain significantly affects the performance of the recombinant strain. Normally, despite strain variation, polyploid, wild-type *S. cerevisiae* is a better ethanol producer than the haploid strain, which is generally used in the laboratory (Brandberg et al. 2004; Li et al. 2015; Sonderegger et al. 2004; Yamada et al. 2011). Although the genetic background issue is complex, basic selection principles are feasible. By evaluating strains for their glucose-fermenting power, stress tolerance, and the ability to metabolize pentose, we can select a strain suitable for use as the chassis cell in lignocellulosic ethanol production (Li et al. 2015).

**Fig. 1:**
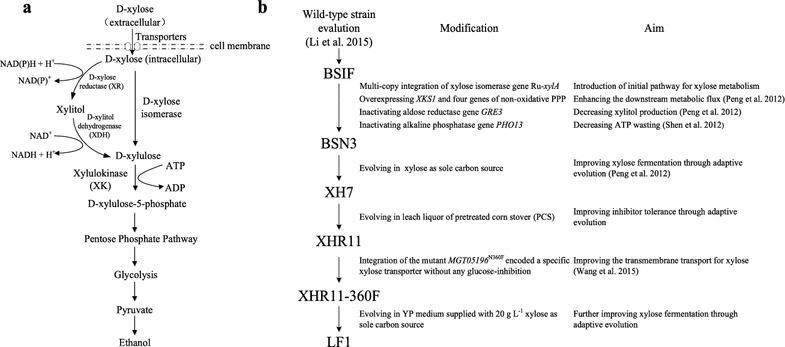
The schematic diagram of xylose metabolism (**a**) and strain parentage (**b**)

The following two heterologous initial xylose metabolic pathways (Fig. 1a) were introduced into an *S. cerevisiae* strain: the XR-XDH pathway, which is composed of xylose reductase (XR) and xylitol dehydrogenase (XDH), and the XI pathway, which is composed only of xylose isomerase (XI). For the XR-XDH pathway, xylose is first converted to xylitol by XR, which has a higher affinity for NADPH than NADH. Then, xylitol is oxidized into xylulose by XDH, which depends exclusively on NAD^+^ (Ho et al. 1999; Peng et al. 2012; Wang et al. 2004). The imbalance in redox metabolism caused by the different coenzyme preferences between XR and XDH result in the accumulation of the byproduct xylitol and a lower ethanol yield; cofactor engineering resulted in limited improvement (Hou et al. 2009a; Zha et al. 2012; Zhang et al. 2010). For the XI pathway, since xylose is directly isomerized to xylulose with no coenzyme participation, incorporating this pathway is a direct and effective strategy for initiating xylose metabolism in *S. cerevisiae* (Demeke et al. 2013; Diao et al. 2013; Zhou et al. 2012). Highly efficient XI activity is a prerequisite for rapid and efficient xylose fermentation in *S. cerevisiae* (Brat et al. 2009; Kuyper et al. 2003; Madhavan et al. 2009; Walfridsson et al. 1996; Zhou et al. 2012). Recently, we screened a bovine rumen metagenomic library and discovered the novel XI gene Ru-*xylA*. Ru-XI exhibited higher activity (1.31 U mg^−1^) in *S. cerevisiae* (Bao et al. 2013; Hou et al. 2016a) than *Pi*-XI (the XI from *Piromyces* sp.), which is a prototypically active XI (Kuyper et al. 2003, 2004).

To compensate for the shortfall in downstream metabolic flux in ethanol production, the xylulokinase Xks1p and the enzymes in the non-oxidative PPP are normally overexpressed in a xylose-utilizing strain (Bamba et al. 2016; Peng et al. 2012; Sharma et al. 2016). Additionally, the inactivation of aldose reductase Gre3p, which is encoded by *GRE3*, to decrease the formation of the byproduct xylitol (Bamba et al. 2016; Fujitomi et al. 2012; Hahn-Hägerdal et al. 2007; Kuyper et al. 2005a; Peng et al. 2012), and the inactivation of alkaline phosphatase Pho13p, encoded by *PHO13*, to reduce ATP waste (Bamba et al. 2016; Lee et al. 2014; Shen et al. 2012; Van Vleet et al. 2008), were also the considered strategies. Normally, xylose is absorbed non-specifically and insufficiently by hexose transporters and is competitively inhibited by glucose in *S. cerevisiae* (Subtil and Boles 2012). Therefore, to address this problem, both wild-type and mutated endogenous and heterologous transporters were screened (Diao et al. 2013; Farwick et al. 2014; Moon et al. 2013; Nijland et al. 2014; Runquist et al. 2009; Shin et al. 2015; Wang et al. 2015, 2016). Moreover, with higher efficiency and/or specificity for xylose, a transporter lacking glucose inhibition may relieve the glucose repression effect in a xylose absorption node, thereby increasing xylose metabolism. Recently, we found the mutant *MGT05196* ^N360F^, which is derived from the *Meyerozyma guilliermondii* transporter gene *MGT05196* and exhibits xylose-specific transport and no glucose inhibition (Wang et al. 2015).

In theory, such rational genetic modifications should endow *S. cerevisiae* with the capacity to ferment xylose; nonetheless, the xylose-fermentation efficiency of the resulting engineered strain was less than expected, indicating the influence of unknown key factors. Additionally, it remains difficult to rationally enhance the tolerance of a xylose-fermenting strain to the inhibitors present in lignocellulosic hydrolysates. Therefore, non-rational, adaptive evolutions were performed in media that contained xylose as the sole carbon source or toxic lignocellulosic hydrolysates to further select for enhanced characteristics both in xylose metabolism efficiency and robustness (Heer and Sauer 2008; Kuyper et al. 2005b; Moysés et al. 2016; Sharma et al. 2016).

The co-fermentation of hexose and pentose is considered important for the effective industrial production of bioethanol. Normally, the concept of co-fermentation usually encompasses only a strain’s ability to ferment xylose and glucose in the presence of both sugars. Therefore, little attention is given to the synchronous fermentation of both sugars. In fact, the obvious hysteresis of xylose fermentation compared with glucose has been extensively observed, manifestations of which exhibit a delay not only with respect to initial xylose utilization and the xylose consumption rate but also the time to maximum ethanol production; in other words, the ethanol production value increases incrementally after glucose exhaustion (Ko et al. 2016; Peng et al. 2012; Reider Apel et al. 2016; Sharma et al. 2016). Therefore, a yeast strain that co-ferments xylose and glucose with the same consumption rate (i.e., synchronously) would be ideal. We believe that the synchronization of sugar fermentation will reduce the apparent fermentation time, improve equipment utilization and employee effectiveness, and play a vital role in the realization of the scaled-up production of second-generation bioethanol.

In the present work, the diploid *S. cerevisiae* strain BSIF, which we have previously shown to be robust and to possess a good background for xylose metabolism (Li et al. 2015), was used as the chassis cell. For rational strain design, two novel proprietary heterologous genes, *MGT05196* ^N360F^ (ZL 2014105263569) (Wang et al. 2015) and the Ru-*xylA* (US 8,586,336 B2, EP 2679686, ZL 201110042170.2) (Bao et al. 2013; Hou et al. 2016a), were integrated into the genome of diploid strain BSIF. Together with other strategies, including the overexpression of the genes *XKS1* (xylulokinase), *TAL1* (transaldolase), *TKL1* (transketolase), *RKI1* (ribose 5-phosphate isomerase) and *RPE1* (ribulose 5-phosphate epimerase) in the non-oxidative PPP and the inactivation of *GRE3* (aldose reductase) and *PHO13* (alkaline phosphatase), a functional xylose metabolic pathway was constructed in BSIF. For non-rational strain design, the recombinant cells were alternately cultured in media using xylose as the sole carbon source or SECS liquor (the leach liquor of steam-exploding corn stover) as stresses for adaptive evolution.

## Results

### Construction of xylose-fermenting *S. cerevisiae* strains

The robust diploid *S. cerevisiae* strain BSIF, which exhibits good performance in fermentation and high tolerance stress (Table 1) (Li et al. 2015), was chosen as a host for the construction of xylose-fermenting recombinant strains for bioethanol production from lignocellulose. Multiple genetic modifications were made to the genome. After each modification, approximately one dozen colonies were evaluated, and the best-growing colony was used in subsequent work. The process is described in detail as follows (Fig. 1b).

**Table 1:** Strains and plasmids used in this study

Strain/plasmid	Genotype/properties	Resource/reference
*Saccharomyces cerevisiae*		
BSIF	Diploid *S. cerevisiae* strain isolated from tropical fruit in Thailand	Laboratory preserved (Li et al. 2015)
BSN3	BSIF derivative; *XKS1p:: loxP*-*TEF1p*, *gre3:: TPI1p*-*RKI1*-*RKI1t*-*PGK1p*-*TAL1*-*TAL1t*-*FBA1p*-*TKL1*-*TKL1t*-*ADH1p*-*RPE1*-*RPE1t*-*loxP*, *pho13:: (TEF1p*-*Ru*-*xylA*-*PGK1t)*_*3*_-*loxP*, three rounds of δ-integration with a fragment containing three tandem expression cassettes of Ru-*xylA* and the selectable marker *loxP*-*KanMX4*-*loxP*	The present work
XH7	Single-colony isolate from adaptive evolution in xylose; based on BSN3	The present work
XHR11	Single-colony isolate from adaptive evolution in SECS liquor; based on XH7	The present work
XH7-N360F	XH7 derivative; *gre3::MGT05196* ^N360F^	The present work
XHR11-N360F	XHR11 derivative; *gre3::MGT05196* ^N360F^	The present work
LF1	Single-colony isolate from adaptive evolution in xylose; based on XHR11-N360F	The present work
Plasmids		
pUG6	*E.coli* plasmid with segment *LoxP*-*KanMX4*-*LoxP*	Güldener et al. (1996)
pJX7	pJFE3; *TEF1p*-*Ru*-*xylA*-*PGK1t*	Bao et al. (2013)
pXIP1	pUC19-based yeast integration plasmid containing the *PHO13*-targeting recombinant arms *PHO13*-*RA1* and *PHO13*-*RA2*, three tandem expression cassettes of Ru-*xylA* and the selectable marker *loxP*-*KanMX4*-*loxP*	The present work
pXIP2	Similar to pXIP1; the recombinant arms *PHO13*-*RA*2 in pXIP1 were replaced with *PHO13*-*RA3* in pXIP2	The present work
pXIδ	pUC19-based yeast integration plasmid containing the δ-sequence-targeting recombinant arms, three tandem expression cassettes of Ru-*xylA* and the selectable marker *loxP*-*KanMX4*-*loxP*	The present work
pJPPP3	pUC19-based yeast integration plasmid containing the *GRE3*-targeting recombinant arms *RA1/2*; an expression cassette for *Sc*-*RKI1*, *Sc*-*TAL1*, *Sc*-*TKL1* and *Sc*-*RPE1*; and the selectable marker *loxP*-*KanMX4*-*loxP*	Peng et al. (2012)
pJPPP4	The *GRE3*-targeting recombinant arms *GRE3*-*RA2* in pJPPP3 were replaced with *GRE3*-*RA3* in pJPPP4	The present work
pUC-N360F	pUC19-based yeast integration plasmid containing *GRE3*-targeting recombinant arms, an overexpression cassette for *MGT05196* ^N360F^, the upstream activating sequence (UAS elements) UAS_CLB_, and the selectable marker *loxP*-*KanMX4*-*loxP*	The present work
YEp-CH	YEp24 derivative; *GAL1p*-*Cre*-*CYC1t*, *TEF1p*-*hygB*-*TEF1t*	Laboratory preserved

Several copies of Ru-*xylA* (Bao et al. 2013) were integrated into the two alleles of the *PHO13* locus and the δ region in turn (Additional file 1: Fig. S1a, b). By replacing the two alleles of the native promoter with the stronger *TEF1p* in situ, the Xks1p gene *XKS1* was overexpressed (Additional file 1: Fig. S1c) (Peng et al. 2011). The four non-oxidative PPP genes *TAL1*, *TKL1, RKI1* and *RPE1* were upregulated by insertion into the two alleles of the *GRE3* locus (Additional file 1: Fig. S1d). Therefore, the genes *PHO13* and *GRE3* were inactivated at the same time (Additional file 1: Fig. S1a, d). After transformant evaluation, the resulting strain BSN3 (Fig. 1b) was selected for the first round of adaptive evolution. The cells were grown under the stress of xylose as a sole carbon source in YP (which was composed of 10 g L^−1^ yeast extract and 20 g L^−1^ peptone) and transferred into fresh medium once the stationary phase was reached. After 360 h of adaptive incubation on xylose, the biomass doubling time (*T*) dropped from ~200 to ~120 min and remained unchanged for several batches. Among the tested isolates, the colony that grew fastest on xylose was named XH7 (Fig. 1b). Then, to enhance the strain’s tolerance to inhibitors, XH7 was transferred into the SECS liquor with urea as a nitrogen source for another round of adaptive evolution. The concentrations of the main components in the SECS liquor are shown in Table 2. After ~900 h of evolution, the culture showed gradually improved growth; the biomass doubling time (*T*) in SECS liquor shortened from ~7 to 3.9 h. The evolved cell populations were spread on SECS liquor-agar plates. The single colony that grew best in SECS liquor was designated XHR11 (Fig. 1b).

**Table 2:** The concentrations of monosaccharides and inhibitors in SECS liquor and different hydrolysates

	SECS liquor	SECS hydrolysate (Hy1)^a^	SPPR hydrolysate (Hy2)^b^
Main monosaccharides (g L^−1^)			
Glucose	9.87 ± 0.07	86.60 ± 0.160	54.94 ± 0.50
Xylose	36.54 ± 0.28	39.09 ± 0.06	23.79 ± 0.19
Solubilized lignin	3.24 ± 0.01	4.13 ± 0.02	–
Main inhibitors			
Weak acids (g L^−1^)			
Formic acid	ND	ND	ND
Acetic acid	4.77 ± 0.14	4.52 ± 0.01	0.00 ± 0.00
Levulinic acid	ND	ND	ND
Furan aldehydes (g L^−1^)			
Furfural	0.37 ± 0.01	0.47 ± 0.01	–
HMF	0.71 ± 0.02	0.66 ± 0.03	–
Total phenolics (mmol L^−1^)	19.63 ± 0.29	26.68 ± 0.61	–

Values are given as the averages and standard deviations of three independent measurements

^a^Hy1 refers to the hydrolysate from SECS supplied by Novozymes

^b^Hy2 refers to the hydrolysate from SPPR supplied by the Shandong Tranlin Group

To further enhance xylose utilization, the mutant transporter gene *MGT05196* ^N360F^, which encodes a xylose-specific, glucose-insensitive transporter (Wang et al. 2015), was introduced into another region of the *GRE3* locus in XHR11 (Additional file 1: Fig. S1d). The resulting strain, XHR11-N360F (Fig. 1b), was then evolved in a medium using xylose as the sole carbon source again. After ~200 h of adaptive evolution, the biomass doubling time (*T*) dropped from ~150 to ~96 min and remained unchanged for several batches. Then, strain LF1 was selected from several isolates due to its faster growth on xylose (Fig. 1b). All the strains used in this study are listed in Table 1.

### Combinatorial strategy improved xylose assimilation and strain tolerance for lignocellulosic hydrolysates

After multiple rational molecular modifications, BSN3 could grow on xylose and convert xylose to ethanol, but its efficiency was not high, similar to previous reports (Demeke et al. 2013; Diao et al. 2013). However, the xylose-evolved strain XH7 was much improved. Compared to BSN3, XH7 exhibited better fermentation performance, especially with respect to xylose consumption and ethanol yield, when the carbon source was either xylose alone or a glucose-xylose mixture (Table 3, Line 1 vs. 2, Line 3 vs. 4). When xylose was the sole carbon source, XH7 consumed all of the 40 g L^−1^ xylose in 26 h, resulting in an ethanol yield of 0.480 g g^−1^ consumed sugar (Table 3, Line 2), which is more than 94% of the theoretical yield. This result was even somewhat higher than the corresponding values in the mixed-sugar fermentation (Table 3, Line 4). In contrast, the biomass yield of XH7 was lower than that of BSN3 (Table 3, Line 1 vs. 2, Line 3 vs. 4). These results indicate that the majority of the consumed xylose in XH7 was used to produce ethanol rather than to maintain cell growth as in BSN3.

**Table 3:** Metabolic characteristics of engineered *S. cerevisiae* strains in batch fermentations of sugars

	Strains	Medium^a^	Fermentation device	Initial inoculum (g DCW L^−1^)	Consumed xylose (g L^−1^)	μ_max_	Product yield (g g^−1^ consumed sugars)						Specific consumption or production rate (g g^−1^ DCW h^−1^)^f^		
							Biomass^b^	Xylitol^c^	Glycerol	Acetate	Ethanol^d^		Xylose	Sugar^e^	Ethanol
1	BSN3	YPX40	Fermentor	0.5	5.62	0.044	0.501	0	0	0	0.210		0.043	-	0.014
2	XH7	YPX40	Fermentor	0.5	40.54	0.141	0.136	0.008	0.019	0.000	0.480		0.569	-	0.310
3	BSN3	YPD80X40	Fermentor	0.5	16.71	0.190	0.110	0.121	0.046	0.033	0.406		0.037	0.382	0.169
4	XH7	YPD80X40	Fermentor	0.5	39.98	0.280	0.091	0.018	0.051	0.015	0.462		0.135	0.792	0.410
5	XH7	Hy1-YP	Shake flask	0.5	23.16	–	0.065	0.016	0.055	0.023	0.390		0.089	0.240	0.060
6	XH7	Hy1-Urea	Shake flask	0.5	18.74	–	0.020	0.013	0.048	0.027	0.371		0.096	0.235	0.080
7	XH7	Hy1-Urea	Shake flask	1.5	34.16	–	0.020	0.011	0.053	0.026	0.400		0.124	0.460	0.184
8	XHR11	Hy1-Urea	Shake flask	1.5	27.45	–	0.032	0.010	0.053	0.034	0.394		0.091	0.425	0.169
9	XH7	YPX40	Shake flask	0.5	41.34	–	–	0.004	0.014	0.003	0.435		0.763	-	0.314
10	XHR11	YPX40	Shake flask	0.5	21.60	–	–	0.002	0.018	0.012	0.374		0.358	-	0.124
11	LF1	YPX40	Shake flask	0.5	41.32	–	–	0.003	0.020	0.012	0.446		1.089	-	0.472
12	XH7	YPD80X40	Shake flask	0.5	33.35	–	–	0.024	0.045	0.016	0.452		0.193	1.057	0.487
13	XHR11	YPD80X40	Shake flask	0.5	25.37	–	–	0.014	0.037	0.012	0.462		0.128	0.913	0.425
14	LF1	YPD80X40	Shake flask	0.5	41.03	–	–	0.007	0.037	0.008	0.475		0.482	1.738	0.819
15	LF1	Hy1-Urea	Shake flask	1.5	36.43	0.034	0.028	0.001	0.045	0.006	0.413		0.227	0.801	0.334
16	LF1	Hy2-Urea	Shake flask	1.5	23.21	0.051	0.035	0	0	0	0.416		0.341	1.292	0.529

Data are the averages of independent triplicate cultivations. All standard errors were less than 5%. The physiological parameters were calculated at either the end of the fermentation or at the time xylose was depleted

^a^The medium was defined in “Methods” section

^b^Biomass yield was calculated based on glucose for cultivation on mixed glucose and xylose or based on xylose for cultivation with xylose as the sole carbon source

^c^Xylitol yield was calculated based on consumed xylose

^d^Glycerol, acetate and ethanol yields were calculated based on all consumed sugars

^e^The specific consumption rate for glucose and xylose in fermentation with mixed sugars

^f^ *DCW* dry cell weight; refers to biomass

**Fig. 2:**
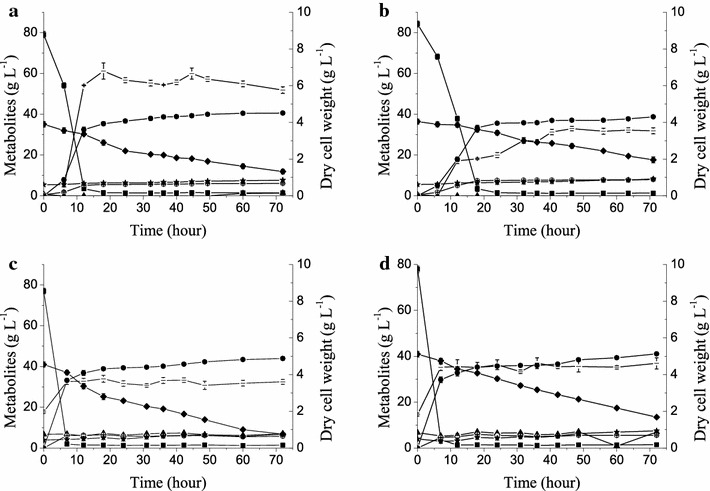
Fermentation characteristics of strains in SECS hydrolysate with different sources of nitrogen and initial inoculum sizes. The experiments were performed in triplicate in SECS hydrolysates supplied by Novozymes (Table 2). Cells were cultured at 30 °C in a shake flask with a rubber stopper and agitation at 200 rpm. Strain XH7 (Fig. 1b; Table 1) **a** with 0.5 g DCW L^−1^ initial biomass in YP (10 g L^−1^ yeast extract and 20 g L^−1^ peptone); **b** with 0.5 g DCW L^−1^ initial biomass in 5 g L^−1^ urea; **c** with 1.5 g DCW L^−1^ initial biomass in 5 g L^−1^ urea; and **d** strain XHR11 with 1.5 g L^−1^ initial biomass in 5 g L^−1^ urea. Symbols: *filled square* glucose; *filled diamond* xylose; *filled triangle* xylitol; *filled circle* ethanol; *open circle* glycerol; *filled star* acetic acid; *dash* biomass (*DCW* dry cell weight)

However, the xylose utilization performance of XH7 worsened in the presence of the inhibitors contained in the SECS hydrolysate (the concentrations of its main components are shown in Table 2). When 0.5 g DCW (dry cell weight) L^−1^ initial cells, which was the same inoculation used in the inhibitor-free medium, were inoculated in YP supplemented with the SECS hydrolysate, XH7 consumed 23.16 g L^−1^ xylose (Fig. 2a; Table 3, Line 5). Because of the costs of industrialization, a cheap nitrogen source, urea, was also tested with initial inoculum sizes of 0.5 or 1.5 g DCW L^−1^; XH7 consumed 18.74 and 34.16 g L^−1^ xylose, respectively (Fig. 2b, c; Table 3, Lines 6, 7). Although richer nutrients and more initial cells improved the fermentation of the hydrolysate, strain performance was still a distinct disadvantage. These results indicate that cell growth was inhibited by the inhibitors derived from the pretreatment and hydrolysis of the lignocellulosic materials.

**Fig. 3:**
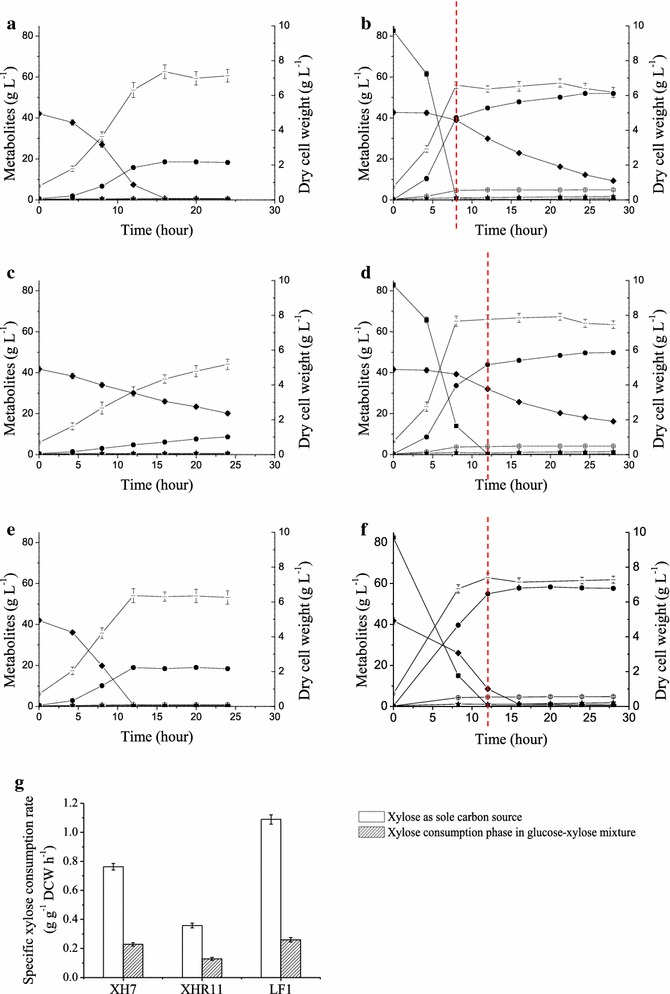
Oxygen-limited fermentation characteristics of strains in xylose and a glucose-xylose mixture in shake flasks. The experiments were performed in triplicate in YP (10 g L^−1^ yeast extract and 20 g L^−1^ peptone) with xylose (*left*) or a glucose-xylose mixture (*right*). Cells with 0.5 g DCW L^−1^ initial biomass were cultured at 30 °C in a shake flask with a rubber stopper and agitation at 200 rpm. Strains (Fig. 1b; Table 1): XH7 (**a**, **b**); XHR11 (**c**, **d**); LF1 (**e**, **f**). Symbols: *filled square* glucose; *filled diamond* xylose; *filled triangle* xylitol; *filled circle* ethanol; *open circle* glycerol; *filled star* acetic acid; *dash* biomass (*DCW* dry cell weight). **g** The specific xylose consumption rate of each strain. In xylose (*blank columns*), the rate was calculated based on the interval from the start to either xylose depletion (**a**, **e**) or the end of fermentation (**c**). In mixed sugars (*shaded columns*), the rate was calculated based on the interval from the glucose-depleted node (*dot line*) either to xylose depletion (**f**) or to the end of fermentation (**b**, **d**)

After an extensive adaptive evolution performed in the presence of inhibitors, the evolved strain XHR11 exhibited better growth in the SECS hydrolysate supplemented with urea. The maximum biomass reached 4.62 g DCW L^−1^, which was more than XH7 achieved (3.77 g DCW L^−1^) (Fig. 2c, d). However, XHR11 consumed less xylose either in the SECS hydrolysate (Fig. 2c, d; Table 3, Line 7 vs. 8) or in the inhibitors-free medium (Fig. 3a vs. c, b vs. d; Table 3, Line 9 vs. 10, Line 12 vs. 13); even glucose utilization was slightly decreased (Fig. 3b, d). These results indicated that adaptive evolution effectively improved the tolerance of the strain to inhibitors but affected the metabolic capacities for these sugars, probably due to some unknown changes caused by toxic stress.

**Fig. 4:**
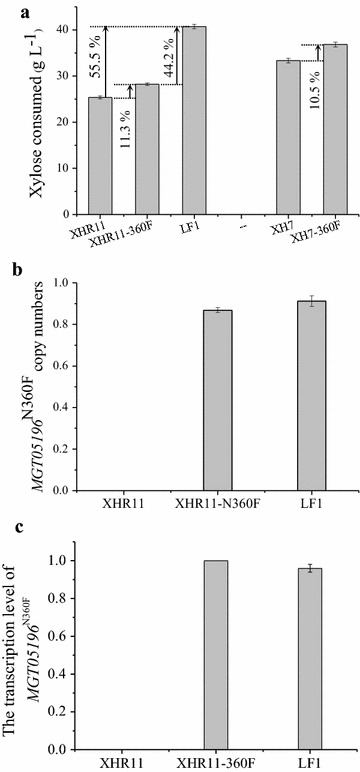
The biography of the transporter mutant *MGT05196* ^N360F^ in strains. Xylose consumption of strains harboring the xylose-specific, glucose-insensitive transporter encoded by the gene *MGT05196* ^N360F^ (Wang et al. 2015) in a glucose-xylose mixture (**a**); transporter gene copy numbers (**b**) and transcription levels (**c**). All experiments were performed in triplicate

In XHR11, the heterologous expression of the transporter mutant *MGT05196* ^N360F^, which encodes a xylose-specific, glucose-insensitive transporter (Wang et al. 2015), together with adaptive evolution on xylose, enhanced xylose consumption by 55.5% in the resulting strain, LF1 (*P* < 0.05) (Fig. 4a). In other words, the xylose metabolic capacity was recovered and the key fermentation parameters were even better in LF1 than in XH7 under inhibitor-free conditions (Table 3, Line 11 vs. 9 and 10, Line 14 vs. 12 and 13; Fig. 3). When xylose was the sole carbon source, the specific xylose consumption rate of LF1 was 1.089 g g^−1^ h^−1^ with an ethanol yield of 0.446 g g^−1^ (Table 3, Line 11), which was greater than 87% of the theoretical yield. In a mixed glucose–xylose fermentation, the ethanol yield was 0.475 g g^−1^ sugars (Table 3, Line 14), which was greater than 93% of the theoretical yield. Moreover, 77.6% of xylose was consumed along with the glucose within 12 h in a glucose–xylose co-fermentation (Fig. 3f), which indicates a higher synchronization in sugar utilization. In conclusion, although LF1 retained the weak glucose utilization inherited from XHR11, its xylose metabolic capacity was rescued, and it obtained a high degree of synchronicity in glucose and xylose utilization (Fig. 3).

**Fig. 5:**
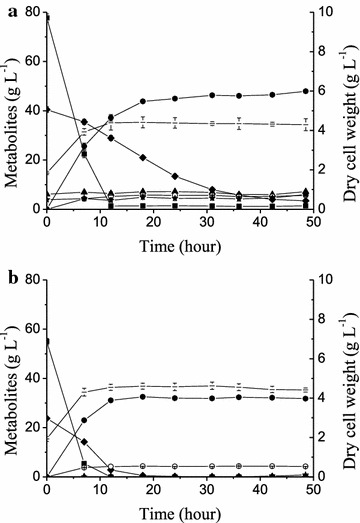
Oxygen-limited fermentation characteristics of strain LF1 in different lignocellulosic hydrolysates. The experiments were performed in triplicate in SECS hydrolysates **a** supplied by Novozymes or SPPR hydrolysates **b** supplied by the Shandong Tranlin Group (Table 2). The cells were cultured at 30 °C in a shake flask with a rubber stopper and agitation at 200 rpm. Symbols: *filled square* glucose; *filled diamond* xylose; *filled triangle* xylitol; *filled circle* ethanol; *open circle* glycerol; *filled star* acetic acid; *dash* biomass (*DCW* dry cell weight)

However, microorganisms with good fermentation performances in the presence of the inhibitors contained in lignocellulosic hydrolysates are candidates for industrial purposes. Therefore, the fermentation capacity of LF1 was tested with the following two different sources of lignocellulosic hydrolysates: SECS and SPPR (sulfite pretreatment papermaking residue) (Table 2; Fig. 5; Table 3, Lines 15, 16). LF1 consumed almost all the sugars in both of the hydrolysates, and both ethanol yields reached over 80.0% of the theoretical yield. In both cases, glucose was depleted within 12 h. However, xylose consumption took longer in SECS (90% xylose in 40 h) than in SPPR (total xylose in 18 h) (Fig. 5). Moreover, the biomass yield in SECS was lower (0.034 g g^−1^) than in SPPR (0.057 g g^−1^) (Table 3, Lines 15, 16). This result is probably due to the toxicity of the SECS hydrolysate, which is higher than that of SPPR (Table 2) and inhibited the cells growth; therefore, the specific xylose consumption and ethanol production rates were also lower (Table 3, Line 15 vs. 16). LF1 and XHR11 were similarly tolerant of the SECS hydrolysate, and both the specific xylose consumption and ethanol production rates were improved compared with those of XH7 (Table 3, Line 7). These results highlight the great potential of LF1 in the practical production of second-generation bioethanol.

### The genetic basis of the efficient xylose metabolism of the evolved strains

As described above, a series of modified strains were derived from the robust diploid yeast (Fig. 1b), and they exhibited an alternation from increase to decrease in terms of xylose metabolic capacity. Therefore, the genetic changes responsible for the xylose assimilation phenotype were preliminarily analyzed.

**Fig. 6:**
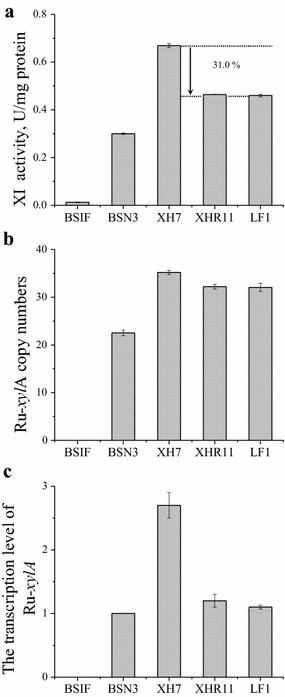
The biography of xylose isomerase (XI) in each strain. Enzyme activities (**a**), copy numbers (**b**) and transcription levels (**c**) of the gene encoding Ru-*xylA* (Bao et al. 2013; Hou et al. 2016a). All experiments were performed in triplicate

The specific activity of xylose isomerase (XI), which is generally considered an important factor (Diao et al. 2013; Hou et al. 2016a), was boosted from 0.30 U mg^−1^ in BSN3 to 0.67 U mg^−1^ in XH7 (Fig. 6a). This improvement was due to the change of copy number of Ru-*xylA* in the genome, which increased from 22.52 ± 0.58 to 35.20 ± 0.42; therefore, the expression level of Ru-*xylA* increased approximately threefold (Fig. 6b, c). A similar phenomenon was also described by Zhou et al. (2012). This increase was possibly due to chromosomal translocations (Pannunzio et al. 2012) and the increased activity of retrotransposons (Robberecht et al. 2013) during adaptive evolution. Unfortunately, inhibitor stress dropped the specific activity of XI by ~31.0% in XHR11 (*P*< 0.05), an effect passed to LF1 (Fig. 6a). We speculated that this impact occurred at the transcriptional level because in comparison with XH7, the copy numbers of Ru-*xylA* in XHR11 and LF1 exhibited no significant difference, but the transcription levels of Ru-*xylA* in these two strains declined significantly, almost to the level in BSN3 (Fig. 6b, c). Even so, the specific activities of xylose isomerase in XHR11 and LF1, which were both ~0.46 U mg^−1^, were still higher than that in BSN3 (Fig. 6a).

The fold changes in the transcript levels of the endogenous genes *XKS1*, *TAL1*, *TKL1*, *RKI1*, and *RPE1* are shown in Table 4. The *XKS1* native promoter was replaced by a stronger in situ promoter, and extra copies of the other genes were integrated into the genome (Additional file 1: Fig. S1c, d); as expected, the genes were overexpressed in BSN3. After adaptive evolution in xylose (Fig. 1b), the transcription levels of *RKI1* and *RPE1* further rose from 14.4 ± 1.3 and 38.0 ± 2.6 to 238.8 ± 15.3 and 51.9 ± 3.6, respectively (in fold changes compared with the original levels in the parent). However, the transcription levels of *XKS1*, *TAL1* and *TKL1* were not significantly different in XH7 compared with those of BSN3. In contrast, evolution in the presence of inhibitors resulted in significantly lower transcription levels of *RKI1* and *RPE1* in XHR11, an effect that persisted in LF1. However, the expression level of *XKS1* in LF1 (21.5 ± 1.5-fold higher than in BSIF) was restored to almost the same level as in XH7 (22.0 ± 1.6-fold higher than in BSIF) due to another round of evolution in the presence of xylose (Fig. 1b). Transaldolase (encoded by *TAL1*) is regarded as the primary rate-limiting step of xylose metabolism in engineered *S. cerevisiae*, and this limitation can be overcome either by overexpressing *TAL1* or by indirectly upregulating it through the deletion of *PHO13* (Xu et al. 2016). In all of the engineered strains in the present work (from BSN3 to LF1), we found that the transcription levels of *TAL1* and *TKL1* were relatively stable (Table 4, Lines 2, 3), which may indicate their positive, conserved role in the xylose fermentation metabolic network (Walfridsson et al. 1997; Xu et al. 2016).

**Table 4:** The gene expression levels of the engineered strains

	Gene	Encoded protein	Fold change (compared with the chassis strain BSIF)				
			BSIF	BSN3	XH7	XHR11	LF1
1	*XKS1*	Xylulokinase	1	20.6 ± 5.2	22.0 ± 1.6	12.3 ± 2.9	21.5 ± 0.5
*2*	*TAL1*	Transaldolase	1	7.7 ± 2.5	10.0 ± 2.0	9.0 ± 0.5	9.4 ± 0.3
*3*	*TKL1*	Transketolase	1	7.3 ± 1.8	6.0 ± 0.6	4.9 ± 0.3	4.6 ± 0.2
*4*	*RKI1*	Ribose 5-phosphate isomerase	1	14.4 ± 1.3	238.8 ± 15.3	146.78 ± 3.5	144.5 ± 0.6
*5*	*RPE1*	Ribulose 5-phosphate epimerase	1	38.0 ± 2.6	51.9 ± 3.6	31.2 ± 0.8	30.0 ± 0.4

Values are given as the averages and standard deviations of three independent measurements

Two modifications contributed to the total 55.5% of xylose consumption increase exhibited by LF1 relative to XHR11. The expression of the glucose-insensitive, xylose-specific transporter Mgt05196p^N360F^ (Wang et al. 2015) led directly to an 11.3% increase in the xylose consumption rate of XHR11 (for XH7, a similar increase of 10.5% was observed). The other 44.2% increase exhibited by LF1 was due to adaptive evolution on xylose (Fig. 4a). Because the copy number and transcription level of *MGT05196* ^N360F^ remained nearly constant (Fig. 4b, c), and strains of XHR11 and XH7 without this transporter were also evolved at the same time but did not exhibit any enhancement in their xylose metabolic capacity in this round of evolution (data not shown), it is evident that this type of transporter was an important positive factor for improving xylose metabolism in *S. cerevisiae*. The transporter probably not only directly enhanced the absorption of xylose but also affected the overall evolutionary trend.

## Discussion

Recently, strains of *S. cerevisiae* engineered for second-generation bioethanol production have been continuously improved, inching closer to industrialization. In general, multiple modifications have been used, including the rational establishment of a xylose metabolic pathway and non-rational adaptive evolution to improve xylose metabolic capacity and inhibitor tolerance (Kim et al. 2013; Ko et al. 2016; Kuyper et al. 2005a; Peng et al. 2012; Van Vleet and Jeffries 2009). Moreover, using robust yeast strains as chassis cells has also proved to be an effective strategy (Demeke et al. 2013; Diao et al. 2013; Li et al. 2015). In the present work, based on our previous study, the *S. cerevisiae* wild-type diploid strain BSIF, which is highly robust and possesses a good genetic background for xylose metabolism, was used as the chassis cell (Li et al. 2015). The following two key novel elements were introduced into the chassis cell: *MGT05196* ^N360F^, which encodes a xylose-specific, glucose-insensitive transporter derived from the *M. guilliermondii* transporter gene *MGT05196* (Wang et al. 2015), and Ru-*xylA*, which encodes a xylose isomerase (XI) with higher activity in *S. cerevisiae* and was derived from a screen of a bovine rumen metagenomic library (Bao et al. 2013). Another highlight in strain construction was the use of multiple adaptive evolutions under xylose and inhibitor stresses.

Overall, these modifications resulted in strain LF1, which has an excellent xylose fermentation capacity in media prepared from both sugars (Fig. 3) and lignocellulosic hydrolysates (Fig. 5). When xylose was the sole carbon source, LF1 consumed all of the 40 g L^−1^ xylose in 12 h with initial inoculum size of 0.5 g DCW L^−1^, and resulted in an ethanol yield of 0.446 g g^−1^. In contrast, CIBTS0735, a similarly engineered strain harboring the xylose isomerase gene *xylA* from *Piromyces* and the transporter gene *GXF1* from *Candida intermedia* (Diao et al. 2013), required 16 h to consume nearly the same amount of xylose with initial inoculum size of 0.63 g DCW L^−1^, and resulted in a lower ethanol yield of 0.412 g g^−1^. Moreover, GS1.11-26, another similarly engineered strain that harbors the xylose isomerase gene *xylA* from *Clostridium phytofermentans* and overexpresses the endogenous transporter gene *HXT7* (Demeke et al. 2013), resulted in a slightly higher ethanol yield of 0.460 g g^−1^ but also required 17 h to ferment less xylose (35 g L^−1^) with a larger initial inoculum size (1.3 g DCW L^−1^). In a glucose–xylose mixture, LF1 consumed 80 g L^−1^ glucose and 40 g L^−1^ xylose in 16 h with an ethanol yield of 0.475 g g^−1^, which was greater than 93% of the theoretical yield. In contrast, the corresponding values for CIBTS0735 (Diao et al. 2013) were 20 h and an ethanol yield of 0.454 g g^−1^ in a fermentation with the same amount of mixed sugars. Additionally, GS1.11-26 (Demeke et al. 2013) required only 13 h to produce an ethanol yield of 0.46 g g^−1^ but fermented less sugar (36 g L^−1^ glucose and 37 g L^−1^ xylose) with a larger initial inoculum size (1.3 g DCW L^−1^) compared with the 0.5 and 0.63 g DCW L^−1^ of LF1 used in the present work (Fig. 5) and CIBTS0735 (Diao et al. 2013), respectively. Other similarly engineered strains did not show improvement in key fermentation parameters, as discussed above (Ko et al. 2016; Romaní et al. 2015; Smith et al. 2014). Additionally, LF1 also performed well in the fermentation of two types of lignocellulosic hydrolysates, even when supplied with the simple nitrogen source urea rather than yeast extract and peptone (Fig. 5). Moreover, LF1 prominently displayed highly synchronized glucose and xylose utilization not only in a mixture of the sugars (with a utilization of 77.6% xylose and glucose depletion at 12 h) but also in SPPR hydrolysate, and ethanol production in LF1 peaked quickly (Fig. 3d vs. f, 5). This characteristic is a significant advantage in second-generation bioethanol production because it will save fermentation time, reduce staff input, and so on.

During approximately 40 years of metabolic engineering in yeast to co-ferment xylose and glucose, researchers have gradually realized that it is easier to establish a xylose metabolic pathway than to improve the efficiency of xylose fermentation and glucose-xylose co-fermentation. Although much research toward this goal has been performed, key factors in this strategy remain unknown. Therefore, non-rational adaptive evolution is generally used for strain engineering (Almeida et al. 2007; Heer and Sauer 2008b; Kuyper et al. 2005b). In the present work, we also focused on the strain breeding, therefore, the contribution for ‘key factors’ exploration maybe not enough.

Increasing the activity of xylose isomerase (XI), which isomerizes xylose directly to xylulose, is considered a good basis for establishing a xylose metabolic pathway in *S. cerevisiae* (Brat et al. 2009; Kuyper et al. 2003; Madhavan et al. 2009; Walfridsson et al. 1996; Zhou et al. 2012). The specific activity of XI in LF1 (0.46 U mg^−1^) was ~31.0% lower than in XH7 (which exhibited the highest activity of the strains in this study, 0.67 U mg^−1^) but was higher than in BSN3 (0.30 U mg^−1^) (Fig. 6a). However, the xylose fermentation capacity of LF1 was much improved compared with that of XH7 and BSN3 (Fig. 3; Table 3). This result implied that maintaining a moderate XI activity has a positive effect on the enhancement of xylose fermentation. The overexpression of Xks1p and four enzymes in the non-oxidative PPP is a common strategy to strengthen xylose metabolism (Bamba et al. 2016; Kuyper et al. 2005a; Peng et al. 2012; Sharma et al. 2016). Previous reports have shown that moderate, rather than extremely high, Xks1p activity is more beneficial for growth and ethanol production from xylose because this enzyme catalyzes an ATP-consuming reaction (Jin et al. 2003; Peng et al. 2011). In the present work (Table 4), the Xks1p (encoded by *XKS1*) expression level fluctuated noticeably. However, the expression level remained relatively high and constant in both improved xylose-fermenting strains, XH7 and LF1. Therefore, such a Xks1p expression level is apparently suitable for xylose metabolism. However, BSN3 also exhibited a similar Xks1p expression level (Table 4), even though this strain has not undergone any adaptive evolution and utilizes xylose poorly (Table 3, Line 1). Obviously, then, a suitable Xks1p activity is not the sole factor governing xylose metabolic capacity. Except for that of ribose 5-phosphate isomerase (encoded by *RKI1*), the expression levels of the enzymes in the non-oxidative PPP showed no significant change (Table 4). After the first round of adaptive evolution under xylose stress, the expression of *RKI1* was upregulated from 14.4 ± 1.3 in BSN3 to 238.8 ± 15.3 in XH7 (in fold change relative to the original chassis, BSIF). Then, inhibitor stress dropped its expression to 146.78 ± 3.5 in XHR11 and remained at 144.5 ± 0.6 in LF1 (Table 4). As discussed above, XH7 and LF1 (but not BSN3 and XHR11) showed improved xylose fermentation, which indicates that ribose 5-phosphate isomerase, rather than the other three enzymes in the non-oxidative PPP, are important for xylose fermentation, as well as other key factors.

The transport issue is another limiting factor for xylose fermentation. Mgt05196p^N360F^, a xylose-specific transporter with no glucose inhibition (Wang et al. 2015), was therefore introduced into the chassis cells. As expected, the transporter enhanced xylose utilization to some extent by simple expression. Moreover, after the Mgt05196p^N360F^-containing strain was evolved in the presence of xylose (Fig. 1b), glucose and xylose utilization efficiency and synchronicity improved (Fig. 3; Table 3). Strain XHR11, which lacked this transporter, did not show any change in its xylose metabolic capacity during the same adaptive evolution, and the copy numbers and transcription levels of *MGT05196* ^N360F^ remained constant in XHR11-360F and LF1. Therefore, we deduced that Mgt05196p^N360F^ absorbed significantly more intracellular xylose, which resulted in stronger xylose stress tolerance in these cells and drove evolution beneficially toward xylose metabolism. Therefore, Xks1p expression recovered in LF1 relative to its parent. However, ribose 5-phosphate isomerase expression did not recover in LF1 (Table 4), which indicates that non-rational adaptive evolution led to random changes in other genes, and the key factors contributing to xylose metabolism must be numerous. An exploration of these unknown factors and their function via inverse metabolic engineering would be a significant undertaking. *S. cerevisiae* maintains high levels of glycolysis and PPP enzymes when glucose is present, and it shifts to respiratory metabolism after glucose is exhausted (Salusjarvi et al. 2008). Because xylose is metabolized through the PPP and glycolysis, its utilization will obviously drop as well. Furthermore, in respiratory metabolism, pyruvate prefers to enter the tricarboxylic acid cycle instead of producing ethanol. Therefore, using a specific transporter to facilitate the synchronized co-fermentation of glucose and xylose is beneficial for their co-utilization.

It was observed that xylose consumption was inhibited by the presence of glucose; therefore, the specific xylose consumption rates in the sugar mixture were lower than when xylose was the sole carbon source (Table 3, line 9 vs. 12, 10 vs. 13, 11 vs. 14) (Demeke et al. 2013; Diao et al. 2013; Peng et al. 2012; Shen et al. 2012), However, the same parameter was still kept lower (Fig. 3g) in the xylose consumption phase (Hou et al. 2009b) after the depletion of glucose from the sugar mixture. We defined such a phenotype—the continued impact of glucose on xylose metabolism after glucose depletion—as the “post-glucose effect” in the present work, which is an underappreciated phenomenon frequently evident in data shown in previous publications (Demeke et al. 2013; Diao et al. 2013; Peng et al. 2012; Shen et al. 2012). Exploration of the mechanisms underlying the post-glucose effect and the discovery of elements to overcome this phenotype will be the focus of future research.

## Conclusions

In the present work, two novel proprietary heterologous genes (*MGT05196* ^N360F^ and Ru-*xylA*) were combined with other multiple genetic modifications and three rounds of adaptive evolution to engineer the diploid *S. cerevisiae* chassis strain BSIF. The final resulting strain of this effort, LF1, consumed all xylose (40 g L^−1^) and glucose (80 g L^−1^) in 16 h with an ethanol yield that was over 93% of the theoretical yield. LF1 also performed well in lignocellulosic hydrolysates, with ethanol yields of over 80.0% of the theoretical yields. Moreover, LF1 exhibited highly synchronous utilization of glucose and xylose. These results highlight the great potential for the practical use of LF1 in the production of second-generation bioethanol. Despite the directed genetic changes detailed in this work, some unknown factors derived from adaptive evolution were also responsible for the superior fermentation performance of LF1. We also outlined the “post-glucose effect” phenomenon. An exploration of its underlying mechanisms remains key.

## Methods

### Media

In the present work, YP was composed of 10 g L^−1^ yeast extract and 20 g L^−1^ peptone. The YPD and YPX were composed of YP amended with glucose and xylose, respectively, in varying concentrations (g L^−1^). For example, YPX40 indicated that 40 g L^−1^ xylose was added to YP. Hy1 and Hy2 refer to the hydrolysates from SECS supplied by Novozymes and SPPR supplied by the Shandong Tranlin Group, respectively. The main components of both hydrolysates are listed in Table 2. Hy1(2)-YP was a mixture of the hydrolysate Hy1 with YP. Hy1(2)-Urea included an additional 5 g L^−1^ urea. SECS liquor referred to the liquid fraction of the steam-exploding corn stover without enzymolysis, which therefore contained less glucose (Table 2).

### Plasmid construction

The chassis cell BSIF was diploid; therefore, to destroy two alleles of *PHO13*, which would benefit xylose metabolism (Bamba et al. 2016; Lee et al. 2014; Shen et al. 2012; Van Vleet et al. 2008), two plasmids were assembled in the plasmid pUC19 as follows: pXIP1/2 (Additional file 1: Fig. S2a) containing two pairs of *PHO13*-targeted recombinant arms, *PHO13*- *RA1* vs. *PHO13*- *RA2* and *PHO13*- *RA1* vs. *PHO13*- *RA3*. Three tandem *TEF1p*-*Ru*-*xylA*-*PGK1t* cassettes, amplified from the plasmid pJX7 (Hou et al. 2014), and the *loxP*-*KanMX4*-*loxP* cassette, amplified from the plasmid pUG6 (Güldener et al. 1996; Peng et al. 2012), were also ligated into the plasmid. Given that the multiple repeats of the δ-sequence in the chromosome could lead to multiple integrated copies of Ru-*xylA* in the genome (Cho et al. 1999; Yamada et al. 2010), a new pair of δ-targeted recombinant arms, *δ*-*RA1* and *δ*-*RA2*, were introduced into pXIP1 instead of the *PHO13*-targeted recombinant arms, resulting in pXIδ (Additional file 1: Fig. S2b). Similarly, inactivating the two alleles of *GRE3* should also benefit xylose metabolism (Kuyper et al. 2005a). The plasmids pJPPP3 (Peng et al. 2012) (which contains individual expression cassettes for four non-oxidative PPP genes and a pair of *GRE3*-targeted recombinant arms, *GRE3*-*RA1/AR2*) and pJPPP4 (which was constructed by replacing the *GRE3*-*RA2* fragment of pJPPP3 with *GRE3*-*RA3*) (Additional file 1: Fig. S2c) were used for this aim.

The specific transporter gene *MGT05196* ^N360F^ (Wang et al. 2015) was inserted into another region of the *GRE3* locus. To achieve this aim, a new pair of *GRE3*-targeted recombinant arms, *GRE3*-*RA4/AR5*, together with the transporter expression cassette *TDH3p*-*MGT05196* ^N360F^-*CYC1t* and the selectable marker *loxP*-*KanMX4*-*loxP*, were ligated into pUC19. Additionally, to heighten expression, a fragment including three tandem sequences of the upstream activating sequence UAS_CLB_ (Blazeck et al. 2012) was also ligated in front of the promoter *TDH3p*. The resulting plasmid, pUC-N360F, is shown in Additional file 1: Fig. S2d.

The relative positions of the introduced gene loci are shown in Additional file 1: Fig. S1. All of the plasmids and primers (with their relevant restriction enzyme sites) used in the present work are listed in Table 1 and Additional file 1: Table S1. respectively.

### Strain construction

All *S. cerevisiae* strains used in this work are listed in Table 1. The strain parentage is shown in Fig. 1b, and diagrams of the relative locations of each genetic manipulation are detailed in Additional file 1: Fig. S1.

Use of the Cre-loxP recombination system, which allows repeated use of a selection marker (Güldener et al. 1996), facilitated construction of the wild-type chassis cell. Accordingly, a new plasmid, YEp-CH, was constructed by the ligation of two fragments, an inducible *GAL1p*-controlled CreA recombinase expression cassette and a dominant hygromycin resistance gene expression cassette, into YEp24. The integration of each fragment into the genome involved several operational steps. The chassis cell was transformed with a *loxP*-*KanMX4*-*loxP*-containing fragment and grown on YPD20 agar plates supplemented with G418 (400 mg L^−1^). After evaluation and verification, the best-growing colony was then transformed with YEp-CH and grown on YPD20 agar plates supplemented with hygromycin (200 mg L^−1^). Then, the transformant was cultured in YP supplemented with galactose (20 g L^−1^) to induce the expression of the CreA recombinase and remove the *KanMX4* cassette. Finally, the YEp-CH plasmid was lost on YPD20 without hygromycin selection. Such steps were cycled (Fig. 1b) in the wild-type chassis cell BSIF (Li et al. 2015).

The *loxP*-*KanMX4*-*loxP*-containing integration fragments were obtained in a different way. The Ru-*xylA* segments were recovered from pXIP1/2 and pXIδ double-digested with EcoRI/SphI. The four genes in the PPP segments were recovered from pJPPP3/4 digested with SmiI. The *MGT05196* ^N360F^ segments were linearized from pUC-N360F digested with SmiI. The *TEF1*p segments with two pairs of *XKS1*-targeted recombinant arms, *XKS1*-*RA1/XKS1*-*RA3* and *XKS1*-*RA2/XKS1*-*RA3*, upstream of *XKS1* were amplified by overlap PCR, which was used to moderately strengthen the expression of *XKS1* (Peng et al. 2011) by replacing its native promoter with a stronger one in two alleles (Additional file 1: Fig. S1c).

### Adaptive evolution

Adaptive evolution was performed under either xylose or inhibitor stress. The cells were cultured at 30 °C and 200 rpm in cotton-plugged 250-mL shake flasks containing 50 mL YPX40 or SECS liquor with YP (pH 6.0), respectively. Cell growth was monitored by measuring the OD_600_ (optical density at 600 nm). Once the stationary phase was reached, a new batch was started by transferring the culture into fresh medium with an initial OD_600_ of 0.5. Evolution was continued until the biomass doubling time (*T*) did not shorten significantly. The biomass doubling time *T* was calculated from OD_600_ during the exponential growth phase using the following equation, as described previously (Peng et al. 2012):

$$ T = \frac{{t_{2} - t_{1} }}{{{ \log }_{2} ({\text{OD}}_{2} /{\text{OD}}_{1} )}} $$

Dozens of colonies were isolated from the evolved strain suspension. Evaluations were performed under the same stress using the BioScreen system (Oy Growth Curves Ab Ltd, Helsinki, Finland) and further verified in shake flasks. Then, the single colony that grew fastest was selected for further use.

### Fermentation

The batch fermentations were performed in either a 1.4-L fermenter with a 1000 mL working volume (Infors AG, Switzerland) or in 250-mL shake flasks with a 50-mL working volume. The different media described above were used as needed. The fermentations were performed at 30 °C and 200 rpm agitation rate, with other parameters of either 0.04 vvm ventilation (the oxygen-limited condition) (Zhang et al. 2010), pH 5.5 in the fermenters, or an initial pH of 6 in the shake flasks with a rubber stopper plug in a syringe needle (the oxygen-limited condition) (Walfridsson et al. 1996). Overnight cultures of a single colony were transferred to 100 mL YPD20 in 250-mL shake flasks at an initial OD_600_ of 0.2 and incubated at 30 °C for another 12 h for inoculations (Li et al. 2015). The data from the independent triplicate cultivations were compared using Student’s *t*-test whenever indicated. A *P* < 0.05 was considered statistically significant.

### Compounds Analysis and Calculation

Biomass was determined by measuring OD_600_ (Eppendorf AG, 22331, Hamburg, Germany) and DCW. Different strains exhibited different coefficients between OD_600_ and DCW (data not shown). The maximum growth rates (μ_max_) are the linear regression coefficients of the *ln*(OD_600_) versus time during the exponential growth phase.

The concentrations of glucose, xylose, xylitol, glycerol, acetate, and ethanol were determined by HPLC (Shimadzu, Japan) with a BIO-RAD Aminex HPX-87H ion exclusion column (300 × 7.8 mm) as previously described (Diao et al. 2013; Hou et al. 2016b; Peng et al. 2012). The mobile phase was 5 mmol L^−1^ H_2_SO_4_ with a flow rate of 0.6 mL min^−1^. The temperature of the column oven was 45 °C. The specific consumption or production rates of xylose, xylitol, glycerol, acetate, and ethanol were calculated as previously described (Peng et al. 2012).

The monosaccharides, weak acid, furfural and HMF in the hydrolysates were measured using an HPX-87H column as described above (Li et al. 2015). The total phenolics were determined using the Folin phenol method, and vanillin was used to prepare the standard curve (Singleton et al. 1999). Solubilized lignin was determined by measuring the absorbance at 320 nm (Li et al. 2015; Tan et al. 2013).

### Measurement of enzyme activities

Overnight cultures were transferred into fresh YPD20 medium with 0.2 initial OD_600_ and cultured at 30 °C. The cells were harvested at OD_600_ 4.0 and washed twice with sterile water and resuspended in 100 mmol L^−1^ Tris–HCl (pH 7.5) with a proteinase inhibitor cocktail (for fungal/yeast cells; Sangon Biotech Co., Ltd., Shanghai, China). Then, the cell-free extract was prepared as the crude enzyme using a Precellys 24 cell homogenizer (Bertin Technologies, France), as previously described (Shen et al. 2012). Protein concentration was measured using an Enhanced BCA Protein Assay Kit (Beyotime Biotechnology, China). The xylose isomerase activity of the crude enzyme was determined at 30 °C by measuring the absorbance change of the coenzymes at 340 nm with a spectrophotometer (Helios Gamma, Thermo Fisher Scientific, Waltham, MA). The 1-mL reaction mixture contained 100 mmol L^−1^ Tris–HCl buffer (pH 7.5), 10 mmol L^−1^ MgCl_2_, 500 mmol L^−1^ xylose, 2 U of sorbitol dehydrogenase (Roche, Boulder, CO), 0.15 mmol L^−1^ NADH, and crude enzyme (Kuyper et al. 2003). One unit of xylose isomerase activity was defined as the amount of enzyme required to oxidize 1 μmol of coenzyme per minute under the conditions of the assay, and the specific activity was expressed in units per milligram of protein (Shen et al. 2012).

### Gene transcription level analysis

Overnight cultures were transferred into fresh YPD20 medium with 0.2 initial OD_600_ and cultured at 30 °C. Cells were harvested at OD_600_ 0.8–1.0. Total RNA was isolated using Trizol reagent (Takara, Japan) and purified using a NucleoSpin^®^ Extract II Kit (Machery-Nagel Corp., Germany). The total RNA (0.25 μg) was used to synthesize the first strand of cDNA in a 10-μL reverse transcription (RT) reaction. Real-time quantitative PCR was performed using the Light Cycle PCR System (Roche Molecular Biochemicals, Germany) and SYBR Green Real-time PCR Master Mix (TOYOBO, Japan). The normalization reference was *ACT1* (Peng et al. 2012). The primers for the quantitative real-time PCR are listed in Additional file 1: Table S1. Real-time quantitative PCR data were analyzed according to the 2^−ΔΔ*CT*^ method (Livak and Schmittgen 2001). All data were the average of values from three separate cultures, and each culture was tested in triplicate. The replicates were compared using Student’s *t*-test whenever indicated. A *P* < 0.05 was considered statistically significant.

### Determination of gene copy number

Cells were washed and resuspended in TE buffer (10 mM Tris–HCl, 1 mM EDTA, pH 7.5) and lysed with a Precellys 24 cell homogenizer (Bertin Technologies, France). The protein was removed using the phenol–chloroform method (Ellington & Jack D. Pollard, in Short Protocols in Molecular Biology, Volume 1, 5th Edition. Edited by Frederick M. Ausubel, Roger Brent, Robert E. Kingston, David D. Moore, J. G. Seidman, John A. Smith, and Kevin Struhl. John Wiley & Sons, Inc.). The genomic DNA was precipitated with ethanol, resuspended in water, and then used to analyze the gene copy number via quantitative real-time PCR. The normalization reference was *ACT1*. The copy number was calculated according to the following equation: plasmid copy number = 2^CT(*act1*)^/2^CT(*xylA*)^ (Lee et al. 2006; Shen et al. 2012).

## Additional file

**Additional file 1.** **Table S1**. Primers used in the current study. **Figure S1**. The relative locations of the introduced genes in each genomic locus. **Figure S2**. The physical maps of the plasmids.

## Abbreviations

XI: xylose isomerase
XR: xylose reductase
XDH: xylitol dehydrogenase
PPP: pentose phosphate pathway
SECS: steam-exploding corn stover
SPPR: sulfite pretreatment papermaking residue
SECS liquor: the leach liquor of steam-exploding corn stover
YP: yeast extract and peptone
DCW: dry cell weight
g: grams
L: liter
h: hours
OD or OD_600_: optical density at 600 nm
RT-qPCR: quantitative reverse-transcription PCR
qPCR: quantitative real-time PCR
