# Supplementary material for: Engineering a wild-type diploid Saccharomyces cerevisiae strain for second-generation bioethanol production
Source: Bioresour Bioprocess. 2016 Nov 24;3(1):51. doi: 10.1186/s40643-016-0126-4 (PMC5122614; doi:10.1186/s40643-016-0126-4)
Supplement: Supplementary file 1 — Additional file 1. Table S1. Primers used in the current study. Figure S1. The relative locations of the introduced genes in each genomic locus. Figure S2. The physical maps of the plasmids. [file 40643_2016_126_MOESM1_ESM.docx]

Supporting Information

**Engineering a wild-type diploid** ***Saccharomyces cerevisiae* strain for second-generation bioethanol production**

Hongxing Li^1,2^^†^, Yu Shen^1†^, Meiling Wu^1^, Jin Hou^1^, Chunlei Jiao^1^, Zailu Li^2^, Xinli Liu^2^, Xiaoming Bao ^1,2*^

^1^ State Key Laboratory of Microbial Technology, Shandong University, Jinan, 250100, China

^2^ Shandong Provincial Key Lab. of Microbial Engineering, Qi Lu University of Technology, Jinan 250353, China

Authors and email list:

Hongxing Li: lihongxing1111@163.com

Yu Shen: shenyu@sdu.edu.cn

Meiling Wu: meilingwu2013@163.com

Jin Hou: houjin@sdu.edu.cn

Chunlei Jiao: jiaochunlei2010@126.com

Zailu Li: [Zl.l2003@126.com](mailto:Zl.l2003@126.com)

Xinli Liu: vip.lxl@163.com

*Correspondence: Xiaoming Bao: [bxm@sdu.edu.cn](mailto:bxm@sdu.edu.cn)

^†^ To be considered as joint ﬁrst authors.

## Table S1. Primers used in the current study.

| Primer | Sequence 5′→3′ | Description |
| --- | --- | --- |
| Amplification of gene fragments | |  |
| RAGRE33s | CTAGTGGCCTATGCAGGCTACCGTTTATTCGATG | Cloning the recombinant arm *GRE3-RA3* for integration targeting to *GRE3* |
| RAGRE33a | TTTCCTGCAGGATTTAAATCAAGTCCCATATCGCT |  |
| RAPHO131s | CCGGAATTCGATGTGACATCTTTACTATT | Cloning the recombinant arm *PHO13-RA1* for integration targeting to *PHO13* |
| RAPHO131a | CGCGGATCCTGCGTCAATTACAATTGAAA |  |
| RAPHO132s | GCCGTGATCAATCGGTTTATAACAAGCAGA | Cloning the recombinant arm *PHO13-RA2* for integration, targeting to *PHO13* |
| RAPHO132a | ACATGCATGCTTGAGCAATCTCCTTATTGG |  |
| RAPHO133s | GCCGTGATCACGTTTCTGTTCGATTGTGAT | Cloning the recombinant arm *PHO13-RA3* for integration, targeting to *PHO13* |
| RAPHO133a | ACATGCATGCCAATACCAAACGAAGCAAAC |  |
| pXIPkans | TCAGAATTCGATCCTGCAGGAGTGGATCCCGTACGCTGCAGGTCGA | Cloning the expression cassette *loxP-KanMX4-loxP* |
| pXIPkana | GAGGCATGCACTGTCGACACGTGATCACACTAGTGGATCTGATATC |  |
| pXIPxylAs | ACATACCCTGCAGGACTGGATCCACAATGCATACTTTGTACGT | Cloning the expression cassette *TEF1p-Ru-xylA-PGK1t* |
| pXIPxylAa | CCGGAATTCGGAAGATCTTAACGAACGCAGAATTTTCG |  |
| RAδ1s | CCGGAATTCTGTTGGAATAAAAATCCAC | Cloning the recombinant arm *δ–RA1*for integration, targeting to δ-sequence |
| RAδ1a | CGCGGATCCCCTATTACATTATCAATCC |  |
| RAδ2s | ATAGGGCCCAACGGAATGAGGAATAATC | Cloning the recombinant arm *δ-RA2* for integration, targeting to δ-sequence |
| RAδ2a | ACCGGCATGCTGAGAAATATGTGAATGTTG |  |
| XKkans1 | aataaaaaaggaaatatttaggggatcaagaccattattCAGCTGAAGCTTCGTACGCTG | Replacing the native promoter of *XKS1* by *TEF1p* |
| XKkans2 | aaacaaaaacaaccccgatttaataacattgtcacagtaCAGCTGAAGCTTCGTACGCTG |  |
| XKkana | GCATAGGCCACTAGTGGATCTG |  |
| XKTEFs | CTAGTGGCCTATGCACAATGCATACTTTGTACGTTCAAAAT |  |
| XKTEFa | aacctctcttgtctgtctctgaattactgaacacaacatTTTGTAATTAAAACTTAGATT |  |
| CLBs | CGCGGATCCAGTGGAATTATTAGAATGACC | Cloning the fragment of UAS_CLB_ |
| CLBa | ATACCCTGCAGGGGAAGATCTGGACAGGCACCGAAGTTCA |  |
| TDH3ps | GTTCGCGATATGGATGAACTTCGGTGCCTGTCCAGATCTAAAAACACGCTTTTTCAGTT | Cloning the sequence of *TDH3p–CYC1t* |
| CYC1ta | ATGATTACGCCAAGCTTGCATGCCTGCAGGGGAAGATCTGGCCGCAAATTAAAGCCTTC |  |
| N360Fs | TTAGTTTCGAATAAACACACATAAACAAACAAATCTAGAATGTCGTCGAATGAGCAGGT | Cloning the open reading frame of *MGT05196(N360F)* |
| N360Fa | GTGAATGTAAGCGTGACATAACTAATTACATGACTCGAGTCAAACCCTTTCGGCTTCGT |  |
| RAGRE33ꞌs | CCCCACTTTTGCCTGAATTTAAATAGGCTACCGTTTATTCGATG | Cloning the recombinant arm *GRE3-RA5* for integration, targeting to *GRE3* |
| RAGRE33ꞌa | GCTAAACAGATCTCTAGACCTAGAGTCAAATAAGGATGGTGT |  |
| RAGRE34s | AGAGCCCGGGCTCCTTCGGTCCTCAATCAT | Cloning the recombinant arm *GRE3-RA4* for integration, targeting to *GRE3* |
| RAGRE34a | ATTTAAATTCAGGCAAAAGTGGGGAATT |  |
|  |  |  |
| Primers for Quantitative Real-time PCR | |  |
| RTXKS1s | TTTACCCCTGCGGAAGAATG | *XKS1* |
| RTXKS1a | GGCAGAGTTGGATGAATGAAAAG |  |
| RTXIs | CTGCTTCCACGATGTAGACCTC | *Ru-xylA* |
| RTXIa | ATAACGCTTGTTGCCGAATACG |  |
| RTRKI1s | CATCAGACAAGGAGGTTCTGCTAA | *RKI1* |
| RTRKI1a | TTCCACCACGCCCACTAAC |  |
| RTTAL1s | TTCTCCTTCGTTCAAGCAGTTG | *TAL1* |
| RTTALa | GTCGGCTTCACCCTTGTAATCT |  |
| RTTKL1s | TTCCTCCTTGGCTGGTCATTT | *TKL1* |
| RTTKL1a | TCCCAACCGTAGGCTTCGTAT |  |
| RTRPE1s | ATATGGCTCTTGTTATGACTGTGGA | *RPE1* |
| RTRPE1a | CTTTCGGGATGGTCTCCTTG |  |
| RTN360Fs | CGGGTTCTGCTTCTTGGACT | *MGT05196*^N360F^ |
| RTN360Fa | CGGTGAGTTGTTGCATGGAC |  |
| RTACT1s | ATGCAAACCGCTGCTCAA | *ACT1* |
| RTACT1a | AGTTTGGTCAATACCGGCAGA |  |

**Figure S1.** The relative location of the introduced genes loci in each genomic locus.

(a) Simultaneous disruption of two alleles of *PHO13* and integration of Ru*-xylA*.

(b) Random integration of Ru*-xylA* at the δ-sequence in the LTR retrotransposon.

(c) Simultaneous destruction of two alleles of *GRE3* and integration of four genes from the non-oxidative PPP and the specific transporter gene *MGT05196*^N360F^.

(d) Replacement of the native promoter of *XKS1* with *TEF1p* at two alleles.

##

**Figure S2.** The physical maps of the plasmids.
